# Physico-Chemical Properties and Oxidative Stability of Fixed Oil from Plum Seeds (*Prunus domestica* Linn.)

**DOI:** 10.3390/biom10020294

**Published:** 2020-02-13

**Authors:** Ivan Savic, Ivana Savic Gajic, Dragoljub Gajic

**Affiliations:** 1 Faculty of Technology in Leskovac, University of Nis, 16000 Leskovac, Serbia; savicivana@tf.ni.ac.rs; 2 Energy Efficiency and Climate Change, EBRD, 11000 Belgrade, Serbia; gajicd@ebrd.com

**Keywords:** oil extraction, solvent polarity, oxidative stability, antioxidant activity, HPLC analysis

## Abstract

Storage of a great amount of plum kernel waste becomes a challenge for food industry. In this work, the plum seed was used as a source of fixed oil that can be an ingredient of commercial products. Soxhlet extraction was carried out using the different solvents, such as *n*-hexane, *n*-heptane, ethyl acetate, acetone, or chloroform:methanol mixture (2:1 *v*/*v*). The highest yield of oil (about 30%) was obtained using *n*-heptane and *n*-hexane, while the lowest yield was obtained using ethyl acetate. The analysis of physico-chemical parameters indicated that all samples of plum seed oil have an exceptional quality. *Schaal oven* test indicated that the fixed oil of plum seed exhibited satisfactory oxidative stability at moderate storage temperatures (up to 65 °C). The composition of phenolic compounds in the oil samples was determined using HPLC method. The most abundant compound of seven identified and quantified phenolic compounds was vanillic acid. The highest content of *β*-carotene (1.67 mg 100 g^−1^ fixed oil) spectrophotometrically determined was in the oil extracted with *n*-hexane. The lowest content of *β*-carotene (1.26 mg 100 g^−1^ fixed oil) was determined in the oil extracted with a mixture of chloroform:methanol (2:1 *v*/*v*). This oil had the highest antioxidant activity (IC_50_ value of 4.35 mg mL^−1^) compared to other oil samples. The antioxidant activity was probably caused by the presence of phenolic compounds. The investigated physico-chemical properties demonstrated that the plum seed oil has a potential for application in the food, cosmetics, and pharmaceutical industry.

## 1. Introduction

Vegetable oils are the main source of the various phenolic compounds, vitamins, tocopherols, pigments, and minerals that have a positive effect on human health [1]. The essential fatty acids (linoleic and *α*-linolenic acid) play an important role in brain function, proper growth, and development, reduction of inflammation and risk factors for the development of heart disease, as well as cancer and arthritis therapy [2,3,4]. The plum seeds have significant amounts of oil (about 30%) [5] that contains various bioactive compounds, including tocols, phenolic compounds [6], proteins, and lipids [7]. Kayano et al. [8] investigated the contribution of some phenolic compounds, caffeoylquinic acid isomers, to the antioxidant activity of *Prunus domestica* Linn.

Among fatty acids, the presence of oleic, linoleic, palmitic, stearic, palmitoleic, arachidonic acids, etc. was confirmed [5,9]. Kamel and Kakuda [10] determined that the content of oleic acid was 52–66% and linoleic acid was 28–35%. They also defined that the content of saturated fatty acids was 5.8–11.3%. Matheus and Oezcan [11] investigated the content of fatty acids and tocopherols in 15 different seeds of *Prunus* spp. from Turkey. The main fatty acids were oleic (43.9–78.5%), linoleic (9.7–37%), and palmitic acid (4.9–7.3%). The total amount of vitamin E-dependent compounds in the oil was varied between 62.9 and 439.9 mg kg^−1^. The most abundant tocopherol was *γ*-tocopherol, while *α*-tocopherol is the main vitamin E-active compound in two species of *P. amygdalus* and in one species of *P. persica*. *α*-tocopherol (9–164.5 mg kg^−1^), *α*-tocotrienol (21.5–41.6 mg kg^−1^), *γ*-tocopherol (1.6–330.2 mg kg^−1^), and *δ*-tocopherol (0–39.1 mg kg^−1^) are included in the composition of fixed oil. According to this study, the content of monounsaturated oleic acid (78.5%) and a small content of saturated palmitic acid (7.3%) were identified in the plum seed oil. Also, they identified linoleic (9.7%), stearic acid (about 1.4%), vaccenic acid (about 1.2%), *α*-linoleic acid (about 0.2%), and arachidic acid (about 0.1%), as well as tocopherols: α-tocopherol (24.1–27.1 mg kg^−1^), *α*-tocotrienol (31.4–41.6 mg kg^−1^), *β*-tocopherol (2.3–4.0 mg kg^−1^), *γ*-tocopherol (133.1–302.1 mg kg^−1^), plastochromanol-8 (1.1 mg kg^−1^), and *δ*-tocopherol (11.4–18.9 mg kg^−1^).

Thanks to this chemical composition, the plum seed oil can be used in food products, but it is also an excellent base for cosmetic products for dry and mature skin. It is easy smeared on the skin without greasy traces and can help the skin damaged by burns [6]. In addition to the commonly used Soxhlet extraction technique, there is an effort to isolate the oil using the ultrasound-assisted and supercritical extraction [5,12]. Until now, *n*-hexane was used mainly for the extraction of plum oil.

Due to the presence of polyunsaturated fatty acids, oils are subject to oxidation. They oxidize to unstable hydroperoxides, which are degraded into the secondary oxidation products (unsaturated ketones, aldehydes, acids, epoxides, etc.) [13]. The oxidation of vegetable oils is an inevitable process and depends on the ingredients that accelerate or slow down these reactions [14]. After consumption, the oxidation process of oils can be continued in vivo. Free radicals and their reaction products cause a variety of harmful effects in the body (gene mutation, oncogenesis, accelerated aging of the body) and many diseases (atherosclerosis, cardiovascular disease, cancer, etc.) [15]. Therefore, it is very important to prevent the oxidation of oils that can occur during their production and storage. The stability of oil can be improved with the addition of antioxidants. Some oils in their composition already contain natural antioxidants (phenolic compounds, carotenoids, and phytosterols) in sufficient quantities so that they can provide the adequate stability of the polyunsaturated fatty acids and the expected shelf life of the product. If oil does not contain enough antioxidants, natural or synthetic antioxidants must be added [16]. During the monitoring of oxidative stability, the oil samples can be stored under ambient conditions from several weeks to several months. This approach to stability testing is not practical for routine oil analyzes so that the accelerated oxidation tests at higher temperatures are used. One of them is the *Schaal oven* test [17].

The main contribution of this work was a valorization of waste in Republic of Serbia caused by plum seeds (*Prunus domestica* Linn.) from the food industries. The different solvents (*n*-hexane, *n*-heptane, acetone, ethyl acetate, and chloroform:methanol mixture (2:1 *v*/*v*)) were used in order to maximize the yield of fixed oils from plum seeds using Soxhlet extraction. Physico-chemical properties and oxidative stability of the obtained samples of oil were investigated. The content of phenolic compounds and antioxidant activity of the oils were determined.

## 2. Materials and Methods

### 2.1. Reagents

In this paper, 96% (*v*/*v*) ethanol (Zorka Pharma, Sabac, Republic of Serbia), *n*-hexane, *n*-heptane, acetone, ethyl acetate, chloroform, toluene, cyclohexane, 2,2-diphenyl-1-picrylhydrazyl (DPPH), methanol (HPLC grade) (Sigma Chemical, St. Louis, MO, USA), methanol, sodium carbonate (Zorka Pharma, Sabac, Republic of Serbia), *Folin-Ciocalteu* reagent, gallic acid (97%) (Merck, Darmstadt, Germany), rutin trihydrate (97%) (Alpha Aesar, Kandel, Germany), vanillic acid, catechin hydrate, syringic acid, epicatechin, chlorogenic acid, caffeic acid (Sigma Chemical, St. Louis, MO, USA), and butylated hydroxytoluene (BHT) (Centrohem, Stara Pazova, Republic of Serbia) were used.

### 2.2. Plant Material

Plum seeds (*Prunus domestica* Linn.) were purchased from Plemic komerc (Osecina, Republic of Serbia). In order to determine the moisture content of plant material, the sample was dried in an oven at 105 °C. The weighing was carried out every two hours after the drying process. The drying procedure was repeated to the constant weight of the sample. The moisture content of plum seed was found to be 1.76% (*w*/*w*). Before the extraction, the plant material was ground in an electric mill to the particle size of 0.3 mm.

The total phenolic content in the initial plant material was determined using the spectrophotometric method described by Singleton et al. [18]. The extract of plum seed was prepared by ultrasound-assisted extraction using 50% (*v*/*v*) ethanol at the liquid-to-solid ratio of 10 mL g^−1^ and a temperature of 50 °C for 30 min. The sample was prepared by mixing 0.1 mL of the extract, 1 mL of the tenfold diluted Folin-Ciocalteu reagent with distilled water, and 1 mL of sodium carbonate (7%, *w*/*w*). The total phenolic content was expressed as milligram of gallic acid equivalents (GAE) per 100 g dry weight (d.w.). The blank solution had the equivalent amount of distilled water instead of sodium carbonate. The absorbance was measured at 760 nm and room temperature (22 °C) in the quartz cuvettes (1 × 1 cm). The incubation of the samples was performed for 90 min. Varian Cary 100 spectrophotometer (Mulgrave, Victoria, Australia) was used for scanning the samples.

### 2.3. Soxhlet Extraction of Plum Seed Oil

Soxhlet extraction of fixed oil from plum seed (50 g) was performed with 500 mL of *n*-hexane, *n*-heptane, acetone, ethyl acetate, or the mixture of chloroform:methanol (2:1 *v*/*v*). These solvents are commonly used for defatting the plant materials, and based on the dielectric constants they cover the broad spectrum of polarity. The reason for the use of the combination of chloroform and methanol is due to better solubility of lipids and other ingredients. The extraction was carried out at the boiling point of the solvent for 6 h. After the extraction, the solvent was separated from the oil by evaporation using a rotary vacuum evaporator at 40 °C.

### 2.4. Determination of Sensory Quality of the Oils

The oils (20 mL) were transferred into a glass beaker and tempered at about 50 °C in an oven for 30 min, after which the sensory properties (color, odor, taste, and aroma) were evaluated.

### 2.5. Physico-Chemical Characterization of the Oils

Physico-chemical properties of plum seed oil, namely: moisture content, density, refractive index (*Abbe* refractometer AR3D, Krüss Optronic, Hamburg, Germany), viscosity (Visco basic plus Fungilab, Hapog, New York, NY, USA) at a speed of 60 rpm using spindle SP-R3, pH (HI 9321, Hanna instruments, Lisbon, Portugal) at 22 ± 2 °C were determined. Acid, saponification, and peroxide numbers were determined in accordance with standard methods described in *Yugoslav Pharmacopoeia V* [19].

### 2.6. Specific Extinction

The extinction coefficients *K*_232_ (conjugated dienes) and *K*_270_ (conjugated trienes) were obtained by measuring the absorbance of 1% of oil solution in cyclohexane at wavelengths of 232 and 270 nm, respectively. The absorbance was measured at room temperature (22 °C) and resolution of 1 nm on a Varian Cary 100 UV-Vis spectrophotometer in a cuvette of 1 × 1 cm.

### 2.7. Oxidative Stability of the Oils

Oxidative stability of the fixed oils was evaluated by measuring the absorbance of the tempered samples according to the *Schaal oven* test. The oil (50 mL) was poured into the open glass vessel and heated for 96 h at 63 ± 2 °C. After that, 1 g of fresh and tempered oils were dissolved in cyclohexane to the mass fraction of 1% (*w*/*w*). The absorbance was measured at the wavelengths of 232 and 270 nm. The oil quality was estimated based on *R*-*value* which represents the ratio of absorbances at these wavelengths (Equation (1)).

(1) $$R-value=\frac{A_{232}^{1\%}}{A_{270}^{1\%}}$$

### 2.8. HPLC Analysis of Phenolic Compounds

The identification and quantification of phenolic compounds in the fixed oils were performed using the HPLC method described by Nour et al. [20]. The separation of the compounds is carried out on a Zorbax Eclipse XDB-C18 column (4.6 × 250 mm, 5 µm) (Agilent Technologies, Santa Clara, CA, USA). The content of phenolic compounds was expressed in milligrams per 100 g of the fixed oil.

### 2.9. Determination of Total Carotenoid Content

The total carotenoid contents in the fixed oils were determined according to the standard method [21]. The oil sample (1.0 g) was accurately weighed and then dissolved in cyclohexane to the mass fraction of 1% (*w*/*w*). The absorbance of the oil sample was measured at a wavelength of 445 nm in relation to the used solvent. The scanning was performed at room temperature (22 °C) and a resolution of 1 nm on the UV-Vis spectrophotometer in a quartz cuvette (1 × 1 cm). The total carotenoid content (*TCC*), expressed over *β*-carotene, was calculated according to Equation (2).

(2) $$TCC\;\left(mg\;kg^{-1}\right)=\frac{383E}{P\cdot C}$$

where are, *E*—a difference in absorbance between the oil and cyclohexane samples, *P*—cuvette size (cm), *C*—the oil concentration (g 100 g^−1^).

### 2.10. DPPH Assay for Determination of Antioxidant Activity

The antioxidant activity of the fixed oils was determined using the DPPH assay. A series of different concentrations of fixed oil in toluene was prepared from the stock solution (1 g of fixed oil dissolved in 10 mL of toluene). The samples were vigorously stirred (20 s on a vortex apparatus) with 1 mL of DPPH radicals solution in toluene (concentration 3 × 10^−4^ mol L^−1^). The incubation of the sample was carried out in the dark at room temperature (22 °C) for 30 min. The absorbances of the samples were measured at 517 nm in relation to toluene. Instead of the oil, the equivalent amount of toluene was added to the sample of the negative control. A synthetic antioxidant BHT was used as a positive control. The inhibition of DPPH radicals in a percentage was calculated according to Savic-Gajic et al. [22].

### 2.11. Statistical Analysis

In order to perform statistical analysis, three replicates of each sample were used. Differences between means were considered statistically significant at the 95% (*p* < 0.05) confidence level.

## 3. Results and Discussion

### 3.1. Total Phenolic Content in the Plum Seeds

The total phenolic content was found to be 198.03 mg _GAE_ 100 g^−1^ d.w. Korekar et al. [23] analyzed the influence of fourteen apricot genotypes on the total phenolic content. The genotypes were grown under similar cultural practices in the Trans-Himalayan Ladakh region. They reported that the total phenolic content was ranged between 92.2 and 162.1 mg _GAE_ 100 g^−1^ d.w. The extracts were performed by treating the powdered apricot seeds (0.5 g) with 20 mL methanol at room temperature for 12 h. The residue was mixed with 20 mL acetone and the process was repeated as described above to extract the lipophilic compounds. The determined content for plum seeds in our study was slightly higher than the available data for apricot seeds.

### 3.2. Oil Extraction Efficiency

Soxhlet extraction of fixed oil from the plum seeds was carried out with the solvents of different polarity. Oil yield and dielectric constants of the used solvents are shown in Table 1.

The oil yield was highly dependent on the solvent polarity. Generally, the application of solvents with a dielectric constant between 6 and 8 leads to a higher oil yield compared with the solvents with a lower or higher dielectric constant [24]. The highest oil yield (about 30%) obtained using non-polar solvents, *n*-heptane and *n*-hexane, was probably due to the extraction of non-lipid compounds [25]. It can be attributed to the amphipathic nature of triglycerides that have both polar and non-polar components. A slightly lower yield (about 27%) was obtained using a medium polarity solvent, chloroform:methanol mixture (2:1 *v*/*v*) and acetone. The lowest yield of 23.5% was obtained using ethyl acetate. The results indicated that should be careful with the choice of solvent because the increase in solvent polarity can limit lipid solubility and lead to hydrolysis of some lipids [24]. The yield of the extracted oil is affected not only by the plum variety and the solvent, but also pre-treatment of the plant material and extraction techniques. Górnas et al. [26] extracted the plum seeds oil by stirring the plant material and *n*-hexane on a vortex apparatus for 1 min and then by ultrasonic treatment (5 min, 35 °C) and centrifugation at 21 °C for 5 min. In that case, the yield of plum seed oil was 35%. The efficiency of Soxhlet extraction of oil from “Stanley” plum seed was 41% using petroleum ether as the solvent [27]. After 6 h of Soxhlet extraction with *n*-hexane, the yields of oil from the plum seeds of varieties “Fezelemanani” and “Famusa” were 29.82% and 25.5%, respectively [5].

### 3.3. Sensory Quality of the Oils

According to the Quality Regulations (2013) [28], the oils with an indication of the raw material must have a pleasant taste and aroma peculiar to the raw material without foreign odor. The oils extracted with non-polar solvents (*n*-hexane and *n*-heptane) were a pale yellow color, while the oils obtained with polar solvents (ethyl acetate, chloroform:methanol mixture (2:1 *v*/*v*), and acetone) were slightly darker and muddier. The samples of oil were neutral without foreign odor. Due to these sensory characteristics, the oils obtained by non-polar solvents could be commercially applied.

### 3.4. Physico-Chemical Characterization of the Oils

The oil quality was evaluated based on color, odor, moisture content, density, refractive index, viscosity, pH value, free fatty acid content, peroxide value, and results of chromatographic analysis. Some of physico-chemical characteristics of the fixed oils are given in Table 2. The increase of moisture content can lead to the accelerated aging and oxidation of the oil, the formation of acidic products, the growth and development of microorganisms. Knowing the moisture content in the oil is very important because it influences the oil treatment process. Plum seed oil with the lowest moisture content was obtained using *n*-heptane, while the highest moisture content was noticed in the case of acetone. Based on these results, it is expected that the oil obtained with *n*-heptane to be the most stable compared with other oils. The oil density was in a broad range from 0.50 g mL^−1^ (chloroform:methanol mixture (2:1 *v*/*v*)) to 1.10 g mL^−1^ (acetone). The obtained results are comparable with the results of other researchers [26]. The refractive index is commonly used to estimate the purity of the oil [29], which in this study was ranged between 1.47 and 1.48. The small variation in the refractive index may be due to the relative purity of the oil. The refractive index of the plum seed oils is in agreement with the literature data [5]. Knowledge of the rheological properties of the oils can be useful information when evaluating product quality, crystallization of oil mixtures, the stability of the emulsion during storage, etc. [30]. The viscosity of oils was ranged between 115.90 and 183.20 mPas. The lowest viscosity was determined for the fixed oil extracted with *n*-heptane, while the highest value was obtained for the fixed oil extracted with ethyl acetate. The difference in viscosity is the result of fatty acids composition. The oils with a higher content of unsaturated fatty acids (linoleic and linolenic acid) are less viscous and have a higher iodine number [31]. With the increase of free fatty acid content (higher acid number), the viscosity of the oil also increases [30]. Górnas et al. [26] determined the kinematic viscosity of 4.29 mm^2^ s^−1^ for plum seed oil at 40 °C. The pH values of the oils ranged between 3.43 (ethyl acetate) and 4.63 (*n*-hexane) are presented in Table 2. The relatively low pH value indicates the acidity of the oil caused by the presence of fatty acids.

The minimum acid value of 1.41 mg KOH g^−1^ was noticed in the oil extraction with *n*-heptane, while a maximum value of 2.81 mg KOH g^−1^ was determined in oil extraction with ethyl acetate. The content of free fatty acids (% oleic acid) was in the ranges of 0.70–1.40. The acid numbers of extracted fixed oils were in accordance with the legal regulation defined by the Quality Regulations (2006) for edible unrefined oils. According to available regulations, the maximum permitted acid number is 4.00 mg KOH g^−1^. Since the acid numbers were lower than the permitted value, the oils will not be exposed to hydrolytic degradation and will have a longer shelf life. The content of free fatty acids (1.32% oleic acid) in the oil extracted with *n*-hexane for “Stanley“ variety of plum used in this work was slightly higher than the oils of “Fezelemanani” and “Famusa” varieties (0.81% and 0.99%, respectively) [5]. The higher acid number of fixed oil for “Stanley“ variety of plum may be due to poor seed storage conditions. The saponification numbers of oils were significantly different (Table 2). The fixed oil extracted with acetone had the lowest saponification number (180.00 mg KOH g^−1^), while the highest value observed for oil extracted with *n*-hexane (198.00 mg KOH g^−1^). The high saponification number of oil extracted with *n*-hexane indicated that the low-molecular-weight fatty acids were present in large quantities. The lowest peroxide number of 1.82 mmol O_2_ kg^−1^ was determined for the fixed oil extracted with acetone. The fixed oil extracted with ethyl acetate (dielectric constant of 6.0) had the greatest peroxide number of 4.29 mmol O_2_ kg^−1^. In all cases, the peroxide number was not more than 10.00 mmol O_2_ kg^−1^, so that the fixed oils can be considered of good quality and suitable for human consumption. The obtained results indicated that the peroxide number was increased with decreasing solvent polarity. Also, the proposed conditions for the isolation of oil can be considered adequate.

### 3.5. Specific Extinction

The spectrophotometric analysis of plum seed oil can provide information on its quality in terms of secondary oxidation changes (enzymatic or chemical) that occurred during the oil preparation. The extinction coefficients *K*_232_ (conjugated dienes) and *K*_270_ (conjugated trienes), obtained by measuring the absorbance of 1% plum seed oil solution in cyclohexane, are given in Table 3. The obtained values of *K*_232_ and *K*_270_ were in the ranges of 2.4764–3.1352 and 0.6025–1.6606, respectively. By legal regulations, specific extinction is not included in the oil quality criteria. The plum seed oil of “Fezelemanani” and “Famusa” varieties, obtained by Soxhlet extraction with *n*-hexane, had *K*_232_ and *K*_270_ values of 1.71–2.14 and 0.78–0.87, respectively [5]. Based on these values, it was concluded that the oils exhibit good inherent resistance to secondary oxidation.

### 3.6. Oxidative Stability of Plum Seed Oils

The oxidative stability of plum seed oils was monitored based on the concentration of primary and secondary oxidation products expressed over *K*_232_ and *K*_270_ values. The results of *Schaal oven* test for fixed oil samples are depicted in Table 3. Based on the obtained values of *K*_232_ and *K*_270_ for fresh and tempered oil, it can be noticed that the oil tempered at 63 °C during 96 h can be subjected to certain oxidative changes. These changes were manifested by the increase in the content of conjugated dienes and trienes, i.e., the R values of tempered oils relative to their initial values. After tempering, the highest increase of about 1.5-fold in the *R*-*value* was observed for oil prepared with chloroform:methanol mixture (2:1 *v*/*v*), while this increase was smaller for other oils. The results of this study indicated that plum seed oil is resistant to oxidative changes at moderate temperatures. This is most likely due to the presence of bioactive compounds with a pronounced antioxidant activity in the oil. The application of *Schaal oven* test for the evaluation of the oxidative stability for different oils can be found in the literature. A cold-pressed plum seed oil from Turkish territory remained stable for 14 days of storage [32]. The same oil had an induction time of 15.1 h determined by the rancimat method. It was significantly higher compared to the induction period of the oils for mulberry (4.1 h) and cherry seeds (1.3 h). The results of this test indicated the high oxidative stability of plum seed oil.

### 3.7. HPLC Analysis of the Oils

The identification and quantification of phenolic compounds in plum seed oilS were performed using the RP-HPLC-UV method based on a comparison of retention times and UV spectra with available standards (Table 4). Among phenolic acids, the most dominant was vanillic acid (29.09–88.17 mg 100 g^−1^ fixed oil). The highest content of vanillic acid was determined in the oil extracted with acetone. Syringic and chlorogenic acids were identified in all samples. The highest contents of syringic and chlorogenic acids were confirmed in the oils extracted with acetone and ethyl acetate, respectively. The presence of caffeic acid was not identified in the fixed oils extracted with non-polar solvents (*n*-hexane and *n*-heptane). These phenolic acids have a potentially beneficial effect on human health [33]. After vanillic acid, the highest content of rutin (3.44–29.65 mg 100 g^−1^ fixed oil) was quantified in the samples. The lowest content of rutin was determined in the oils extracted with non-polar solvents, while its content increased with increasing solvent polarity. The secondary metabolites from the flavan-3-ol group, catechin hydrate, and epicatechin, were also identified. Catechin hydrate was determined only in the oils extracted with ethyl acetate and chloroform: methanol mixture (2:1 *v*/*v*), while epicatechin was determined in the oils extracted with *n*-hexane and *n*-heptane. The initial plant material had a higher content of phenolic compounds (198.03 mg _GAE_ 100 g^−1^ d.w.) compared with the summarized content of the identified phenolic compounds in the oils. This fact was expected because the phenolic compounds are well soluble in the polar solvents, such as the used solvent for extraction (ethanol). Khallouki et al. [9] identified benzoic acid, *p*-hydroxybenzaldehyde, *p*-hydroxybenzoic acid, vanillin, vanillic acid, gallic acid, syringaldehyde, syringic acid, coniferyl aldehyde, amygdalin, and others in the methanolic extract of plum seeds. They determined that the contents of vanillic and syringic acids were 29 mg and 0.63 mg per 1 kg of plant material, respectively. The reported content of vanillic acid was manifold lower compared to our study, but their content of syringic acid was slightly higher.

### 3.8. Total Carotenoid Content

The total carotenoid content in the oils, expressed over the content of *β*-carotene (mg 100 g^−1^ fixed oil), is shown in Table 4. *β*-carotene is a precursor in the production of vitamin A in the body. The highest *β*-carotene content was determined in the oil extracted with *n*-hexane (1.67 mg 100 g^−1^ fixed oil), while approximately the same content was determined in the oil extracted with acetone (1.65 mg 100 g^−1^ fixed oil). The minimum content of *β*-carotene (1.26 mg 100 g^−1^ fixed oil) was determined in the oil extracted with chloroform:methanol (2:1 *v*/*v*). The yellow color of the plum seed oil is due to the presence of *β*-carotene. These results were significantly higher compared with the literature data for the plum seed oil of “Renkloda Ronnie Donetskiy” and “Tegera” varieties (0.91–3.07 mg 100 g^−1^) [12]. Since *β*-carotene has an ability to protect the cells from free radicals, it is recommended in the prevention of skin aging, cardiovascular disease, and cancer [34]. The daily needs for vitamin A are between 700 and 900 μg [35]. Having this in mind, the plum seed oil can be used for the development of dietary supplements.

### 3.9. Antioxidant Activity of the Oils

The antioxidant activity of plum seed oils was determined using the DPPH assay. IC_50_ values of the fixed oils obtained by using solvents of different polarity are shown in Table 5.

The results of IC_50_ for the sample of BHT is also depicted in order to compare the antioxidant activity of oils with this commercial antioxidant. The antioxidant activity of the fixed oil was decreased in the following order of the applied solvent: chloroform:methanol (2:1 *v*/*v*) > acetone > *n*-hexane > *n*-heptane > ethyl acetate. The IC_50_ value of BHT (0.04 mg mL^−1^) indicated that this synthetic antioxidant had the highest antioxidant activity compared to the other samples. The fixed oils extracted with more polar solvents, chloroform:methanol mixture (2:1 *v*/*v*) and acetone, had lower IC_50_ values of 4.35 and 6.16 mg mL^−1^, respectively. These values indicate that these oils have better antioxidant activity compared to the fixed oils extracted with non-polar solvents. This was be expected because phenolic compounds responsible for antioxidant activity are rather extracted by polar solvents than non-polar solvents. The efficient extraction of phenolic compounds was resulted by using the polar solvents [22]. The oil extracted with ethyl acetate had the lowest antioxidant activity since the highest IC_50_ value of 12.55 mg mL^−1^ was obtained in that case. The antioxidant activity of the extracted oils was due to the presence of the identified phenolic compounds, such as rutin, vanillic, syringic, and chlorogenic acid. Since the plum seeds oils had a satisfactory antioxidant activity, the oxidative stability of polyunsaturated fatty acids can be achieved without the addition of other antioxidants. The antioxidant activity (63.3 mg Trolox 100 g^−1^ oil) of cold-pressed plum seed oil from Turkey was determined using DPPH assay [32]. Khallouki et al. [9] determined the antioxidant activity of methanolic extract of plum seeds and calculated the IC_50_ value of 0.33 mg mL^−1^. The antioxidant activity was higher for methanolic extract compared with the antioxidant activity of oil samples due to the presence of numerous phenolic compounds.

## 4. Conclusions

Soxhlet extraction was successfully applied for the isolation of fixed oils from plum seed using five different solvents. The solvent polarity was varied in order to maximize the oil yield and to investigate their impact on the physico-chemical characteristics of the fixed oil. The plum seed oils have pronounced sensory properties to the original raw material. The oils prepared using non-polar solvents had a clear and pale yellow color, while the oils obtained by polar solvents were slightly darker. Since the more efficient extraction of fixed oils from plum seeds was achieved using non-polar solvents (*n*-hexane and *n*-heptane), this fact can be of significant importance from an economic point of view in the process industry. Further work will be focused on the identification and quantification of fatty acids in the extracted oils because these results are important for the development of dietary supplements and cosmetic products.

## Figures and Tables

**Table 1:** The yield of plum seed oil.

Solvent	Oil Yield [%]	Dielectric Constant of Solvent
*n*-hexane	30.0	1.9
*n*-heptane	30.5	1.9
ethyl acetate	23.5	6.0
chloroform:methanol (2:1 *v*/*v*)	27.3	18.0
acetone	27.6	21.5

**Table 2:** Physico-chemical properties of plum seed oils.

Parameters	*n*-Hexane	*n*-Heptane	Ethyl Acetate	Chloroform:Methanol (2:1 *v*/*v*)	Acetone
moisture content [%]	0.89	0.52	0.65	0.72	0.93
density [g mL^−1^]	0.90	0.80	0.70	0.50	1.10
refractive index	1.47	1.47	1.48	1.47	1.47
viscosity [mPas]	118.20	115.90	183.20	176.00	135.40
pH value	4.63	3.57	3.43	4.06	4.20
acid number [mg KOH g^−1^]	1.63	1.41	2.81	2.24	1.53
saponification number [mg KOH g^−1^]	198.00	184.00	188.00	193.00	180.00
peroxide number [mmol O_2_ kg^−1^]	3.75	2.50	4.29	2.00	1.82

**Table 3:** The extinction coefficient of fresh and tempered oils at 232 and 270 nm.

Solvent	Fresh Oil		Tempered Oil		*R*-*Value*	
	*K*_232_	*K*_270_	*K*_232_	*K*_270_	Fresh Oil	Tempered Oil
*n*-hexane	3.1352	0.8853	4.0115	0.9062	3.5415	4.4267
*n*-heptane	2.6922	0.6096	3.9075	0.6703	4.4163	5.8295
ethyl acetate	2.4764	0.6150	3.6974	0.6654	4.0265	5.5566
chloroform:methanol (2:1 *v*/*v*)	3.0026	1.6606	4.6136	1.7358	1.8081	2.6579
aceton	2.7648	0.6025	3.5124	0.6432	4.5890	5.4609

**Table 4:** The contents of phenolic compounds and *β*-carotene in plum seed oil.

Compound	Concentration [mg 100 g^−1^ Fixed Oil]				
	*n*-Hexane	*n*-Heptane	Ethyl Acetate	Chloroform:Methanol (2:1 *v*/*v*)	Acetone
vanillic acid	32.71	34.78	29.09	46.17	88.17
rutin	3.44	4.32	29.65	22.38	n. d.
catechin hydrate	n. d.	n. d.	0.03	0.02	n. d.
syringic acid	0.02	0.03	0.05	0.05	0.07
epicatechin	0.08	0.07	n. d.	n. d.	n. d.
chlorogenic acid	0.03	0.04	2.95	1.84	0.08
caffeic acid	n. d.	n. d.	0.08	0.11	0.02
*β*-carotene	1.67	1.51	1.44	1.26	1.65

n. d.—not detected.

**Table 5:** Antioxidant activity of the plum seed oils.

Oil	IC_50_ [mg mL^−1^]	Concentration [mg mL^−1^]
*n*-hexane	9.0	0.39–25
*n*-heptane	9.39	0.78–50
ethyl acetate	12.55	0.39–50
chloroform:methanol (2:1 *v*/*v*)	4.35	0.78–50
acetone	6.16	0.39–25
BHT	0.04	0.08–0.25
